# Bone Marrow CX3CL1/Fractalkine is a New Player of the Pro-Angiogenic Microenvironment in Multiple Myeloma Patients

**DOI:** 10.3390/cancers11030321

**Published:** 2019-03-06

**Authors:** Valentina Marchica, Denise Toscani, Anna Corcione, Marina Bolzoni, Paola Storti, Rosanna Vescovini, Elisa Ferretti, Benedetta Dalla Palma, Emanuela Vicario, Fabrizio Accardi, Cristina Mancini, Eugenia Martella, Domenico Ribatti, Angelo Vacca, Vito Pistoia, Nicola Giuliani

**Affiliations:** 1 Department of Medicine and Surgery, University of Parma, 43126 Parma, Italy; valentina.marchica@unipr.it (V.M.); denise.toscani@gmail.com (D.T.); marina.bolzoni@unipr.it (M.B.); paola.storti@unipr.it (P.S.); rosanna.vescovini@unipr.it (R.V.); benedetta.dallapalma@gmail.com (B.D.P.); emanuela_vicario@hotmail.it (E.V.); accardi.fabrizio@gmail.com (F.A.); 2 Center for Autoinflammatory Diseases and Immunedeficiencies, IRCCS “Istituto Giannina Gaslini”, 16147 Genoa, Italy; annacorcione@gaslini.org; 3 Center of Excellence for Biomedical Research (CEBR), University of Study of Genoa, 16132 Genoa, Italy; elisaferretti@gaslini.org; 4 Hematology, “Azienda Ospedaliero-Universitaria di Parma”, 43126 Parma, Italy; 5 “U.O. di Anatomia Patologica, Azienda Ospedaliero-Universitaria di Parma”, 43126 Parma, Italy; cmancini@ao.pr.it (C.M.); emartella@ao.pr.it (E.M.); 6 Department of Basic Medical Sciences, Neurosciences and Sensory Organs, University of Bari, 70124 Bari, Italy; domenico.ribatti@uniba.it; 7 Department of Biomedical Science and Human Oncology, University of Bari, 70124 Bari, Italy; angelo.vacca@uniba.it; 8 Immunology Area, “Ospedale Pediatrico Bambino Gesù”, 00165 Roma, Italy; vito.pistoia@opbg.net

**Keywords:** multiple myeloma, angiogenesis, inflammation, CX3CL1/fractalkine, microenvironment

## Abstract

C-X3-C motif chemokine ligand 1 (CX3CL1)/fractalkine is a chemokine released after cleavage by two metalloproteases, ADAM metallopeptidase domain 10 (ADAM10) and ADAM metallopeptidase domain 17 (ADAM17), involved in inflammation and angiogenesis in the cancer microenvironment. The role of the CX3CL1/ C-X3-C motif chemokine receptor 1(CX3CR1) axis in the multiple myeloma (MM) microenvironment is still unknown. Firstly, we analyzed bone marrow (BM) plasma levels of CX3CL1 in 111 patients with plasma cell disorders including 70 with active MM, 25 with smoldering myeloma (SMM), and 16 with monoclonal gammopathy of undetermined significance (MGUS). We found that BM CX3CL1 levels were significantly increased in MM patients compared to SMM and MGUS and correlated with BM microvessel density. Secondly, we explored the source of CX3CL1 in MM and BM microenvironment cells. Primary CD138^+^ cells did not express CXC3L1 but up-regulated its production by endothelial cells (ECs) through the involvement of tumor necrosis factor alpha (TNFα). Lastly, we demonstrated the presence of CX3CR1 on BM CD14^+^CD16^+^ monocytes of MM patients and on ECs, but not on MM cells. The role of CX3CL1 in MM-induced angiogenesis was finally demonstrated in both in vivo chick embryo chorioallantoic membrane and in vitro angiogenesis assays. Our data indicate that CX3CL1, present at a high level in the BM of MM patients, is a new player of the MM microenvironment involved in MM-induced angiogenesis.

## 1. Introduction

Tumor-promoting inflammation and angiogenesis are hallmarks of cancer [1]. Multiple myeloma (MM) is a hematological malignancy characterized by the tight dependence of malignant plasma cells (PCs) on the bone marrow (BM) microenvironment [2,3]. These relationships activate the production of several pro-inflammatory cytokines, chemokines and growth factors supporting MM cell survival, bone destruction, and an increased BM angiogenesis [4,5,6,7] that typically correlates with disease stage and prognosis [8,9,10].

C-X3-C motif chemokine ligand 1 (CX3CL1), also known as fractalkine, is a protein of 373 amino acids with three main domains: Chemokine, mucin-like stalk, and transmembrane [11]. In particular, the mucin-like domain contains a cutting site allowing the metalloproteases ADAM metallopeptidase domain 10 (ADAM10) and ADAM metallopeptidase domain 17 (ADAM17) to cleave and release the protein in a soluble form [12,13]. Indeed, CX3CL1 can be also found as a membrane-bound protein. By binding to its only receptor C-X3-C motif chemokine receptor 1 (CX3CR1), CX3CL1 plays a role in both chemotaxis and cell adhesion, especially in endothelial cells (ECs) [14,15]. In both cancer and inflammation processes, CX3CL1 has been shown to stimulate chemotaxis, recruiting cells that expressed CX3CR1, such as natural killer (NK) cells, dendritic cells (DCs), and monocytes [16,17,18]. Moreover, CX3CL1 induces angiogenic potential in CX3CR1-expressing monocytes. CX3CL1 activates the SRC/focal adhesion kinase (FAK) signaling pathway in cancer cells regulating their migration and invasion [19,20] and facilitates bone metastasis of CX3CR1-expressing tumor cells [21,22].

The pathophysiological role of the CX3CL1/CX3CR1 axis in the interaction between tumor cells and the microenvironment has been demonstrated in several solid tumors, such as breast and prostate and in different types of B cell lymphomas and chronic lymphocytic leukemia [11]. A possible involvement of CX3CR1 in the cross talk between neoplastic B cells and the tumor microenvironment has been proposed since CX3CL1 and CX3CR1 are co-expressed on different types of B malignant cells. Recently, it has also been reported that the CX3CL1/CX3CR1 axis could be involved in the stimulation of MM cell adhesion to the extracellular matrix and the production of soluble factors that promote osteoclast differentiation [23]. However, the pathophysiological role of the CX3CL1/CX3CR1 axis in the MM microenvironment is still unexplored. In the present study, we analyzed the BM levels of CX3CL1 in a large cohort of patients with MM and indolent monoclonal gammopathies, and we identified the CX3CL1/CX3CR1 axis as a new player of the vascular microenvironment involved in MM-induced angiogenesis.

## 2. Results

### 2.1. CX3CL1 Bone Marrow Plasma Levels Increase across the Progression of Multiple Myeloma Disease

**Figure 1:**
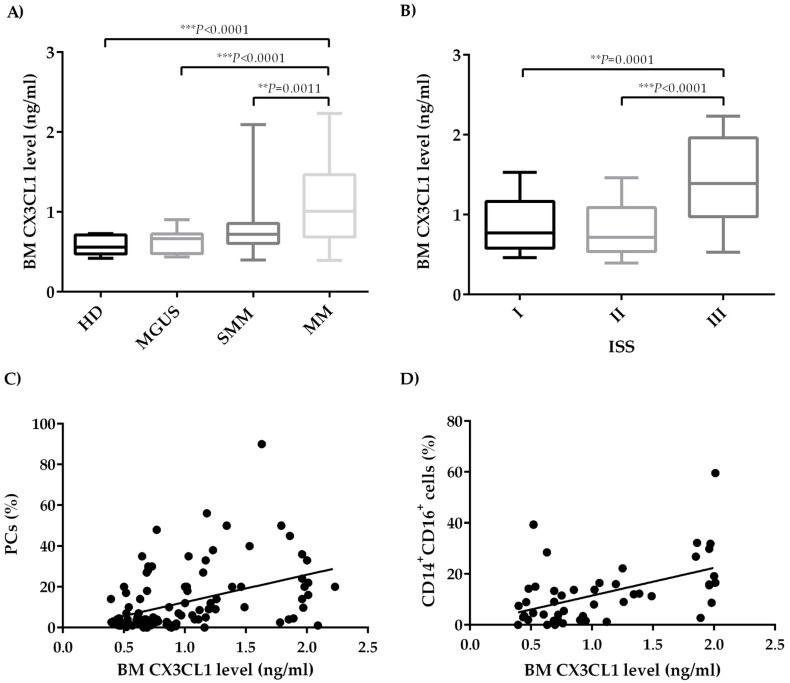
BM levels of CX3CL1 in patients with monoclonal gammopathies. (**A**) Box plots represent the median levels of CX3CL1 (ng/ml) evaluated in BM plasma obtained from patients with MGUS (n = 16), SMM (n = 25), MM (n = 70), and HD (n = 10) (*P* value calculated by Mann–Whitney test). (**B**) Box plots represent the median levels of BM CX3CL1 in patients with active MM grouped by the ISS stage I (n = 17), II (n = 20), and III (n = 33) (*P* value calculated by Mann–Whitney test). (**C**) Scatter plots showing the correlation between CX3CL1 BM plasma levels and percentage of BM PCs obtained from the BM aspirate of 105 patients with monoclonal gammopathies by flow-cytometry analysis (*** *P* < 0.0001, r = 0.44 calculated by Spearman’s correlation). (**D**) Scatter plots show a significant positive correlation between CX3CL1 levels in BM plasma obtained from 48 patients with monoclonal gammopathies and percentage of CD14^+^CD16^+^ monocytes evaluated by flow cytometry (** *P* = 0.0006, r = 0.48 calculated by Spearman’s correlation). CX3CL1—C-X3-C motif chemokine ligand 1; MUGS—monoclonal gammopathy of undetermined significance; HD—healthy donors; SMM—smoldering myeloma; MM—multiple myeloma; BM—bone marrow; PC–plasma cell; ISS—International Staging System.

We firstly evaluated BM CX3CL1 levels in our cohort of 111 patients, including 16 patients with monoclonal gammopathy of undetermined significance (MGUS), 25 with smoldering myeloma (SMM), and 70 with active MM, and 10 healthy donors (HD) as controls. BM CX3CL1 levels significantly increased across the groups of subjects analyzed (median range BM CX3CL1 level in HD = 0.560 (0.420–0.730) ng/ml; MGUS = 0.665 (0.435–0.902) ng/ml; SMM = 0.720 (0.398–2.090) ng/ml; MM = 1.008 (0.394–0.230) ng/ml) (*P* < 0.0001, Kruskal–Wallis test). Specifically, CX3CL1 levels were significantly increased in the BM plasma of MM patients compared to HD, MGUS, and SMM patients (*P* < 0.0001, *P* < 0.0001, and *P* = 0.0011 respectively, Mann–Whitney test) (Figure 1A). Based on the International Staging System (ISS), we found that MM patients with ISS III had higher CX3CL1 BM levels as compared to those with ISS I and ISS II, as shown in Figure 1B (*P* = 0.0001 and *P* < 0.0001, Mann–Whitney test) (median range BM CX3CL1 level in ISS I = 0.770 (0.46–1.530) ng/ml; II = 0.71 (0.394–1.460) ng/ml; and III = 1.38 (0.53–2.23) ng/ml). Moreover, BM soluble CX3CL1 levels were positively correlated with the percentage (%) of BM PCs checked by flow cytometry in the BM aspirates (*P* < 0.0001, r = 0.44, Spearman’s correlation) (Figure 1C). On the other hand, any significant correlation was not observed between the BM CX3CL1 plasma levels with the presence of osteolytic lesions (*P* = 0.34, not significant NS, Supplemental Figure S1A) or the presence of high bone disease (HBD) compared to low bone disease (LBD) (*P* = 0.78, NS) in MM patients (Supplemental Figure S1B).

Moreover, since the monocytic subset CD14^+^CD16^+^ shares both pro-osteoclastogenic and angiogenic properties and increased in MM patients compared to patients with asymptomatic disease [24,25], we analyzed the possible correlation between BM CX3CL1 levels and CD14^+^CD16^+^ cells. Interestingly, we found that BM CX3CL1 levels positively correlated with the percentage of BM CD14^+^CD16^+^ monocytes (Figure 1D) (*P* = 0.0006, r = 0.48, Spearman’s correlation) in the sub-cohort of the patients analyzed (7 MGUS, 10 SMM, and 31 MM). A multivariate analysis confirmed that CX3CL1 BM levels significantly correlated with the percentage of both PCs (*P* = 0.003) and BM CD14^+^CD16^+^ monocytes (*P* = 0.0001).

### 2.2. Bone Marrow CX3CL1 Levels Correlate with Bone Marrow Vascularization in Multiple Myeloma Patients

**Figure 2:**
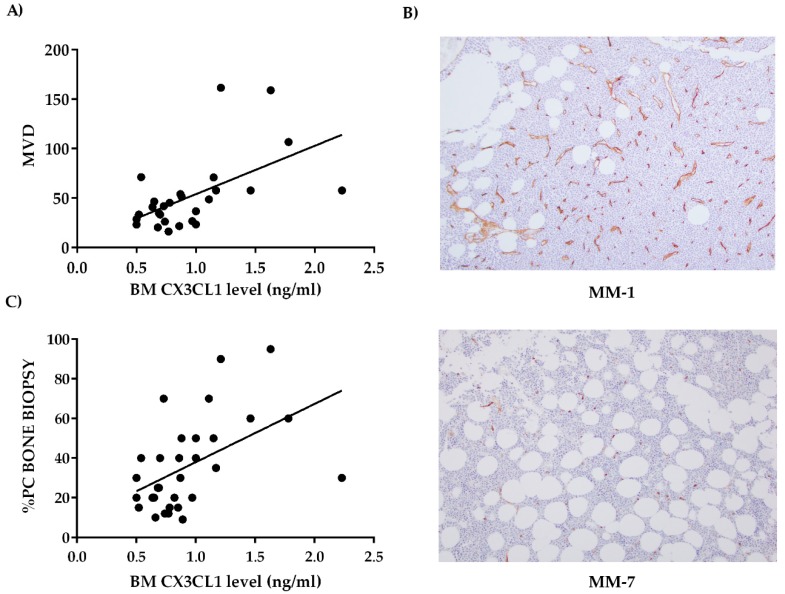
BM CX3CL1 correlates with MVD in MM patients. (**A**) The graph shows a significant positive correlation between CX3CL1 levels in BM plasma obtained from 27 MM patients and MVD, evaluated as CD34^+^ cells/mm^2^ by immunohistochemistry (IHC) (** *P* = 0.0019, r = 0.57 calculated by Spearman’s correlation). (**B**) The pictures show the BM CD34 staining in IHC of two representative MM patients with high (MM-1) and low (MM-7) BM CX3CL1 levels. Original magnification 10×. (**C**) The graph displays the correlation between CX3CL1 BM plasma levels and percentage of PCs by IHC from bone biopsies obtained from 27 MM patients (** *P* = 0.0013, r = 0.58 calculated by Spearman’s correlation). MVD–microvessel density; IHC—immunohistochemistry.

On the basis of the well-known involvement of CX3CL1 in angiogenesis [26], we sought to determine whether MM BM CX3CL1 levels could be related to BM angiogenesis. Interestingly, the number of CD34^+^ vessels significantly positively correlated with BM CX3CL1 levels in MM patients (*P* = 0.0019, r = 0.57, Spearman’s correlation) (Figure 2A). Representative immunohistochemical CD34 staining of MM patients with high and low BM CX3CL1 levels is reported in Figure 2B. Similar to that observed with BM aspirates, we also found that BM soluble CX3CL1 levels significantly positively correlated with the number of BM PCs in bone biopsies (Figure 2C) (*P* = 0.0013, r = 0.58, Spearman’s correlation). Multivariate analysis showed that BM microvessel density (MVD) significantly correlated with the number of PCs (*P* = 0.0001) and with the BM CX3CL1 levels even though statistical significance was not reached (*P* = 0.08).

### 2.3. CX3CL1 Is Released by Endothelial Cells in the Presence of Multiple Myeloma Cells: Role of Human Tumor Necrosis Factor Alpha

**Figure 3:**
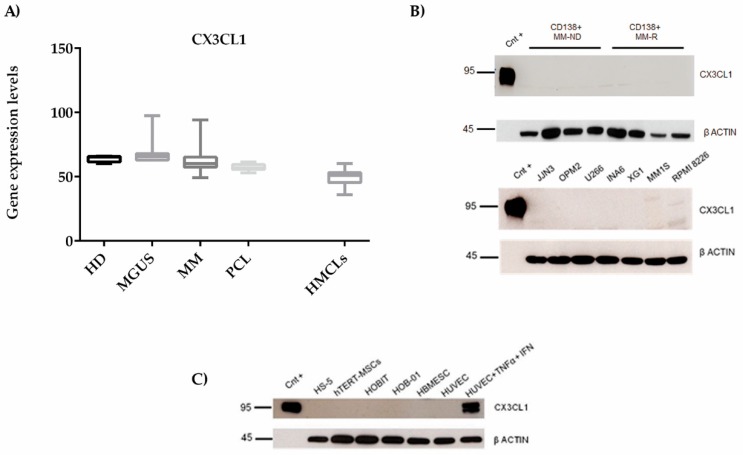
(**A**) CX3CL1 expression by MM cells. Box plots of the expression levels of *CX3CL1* mRNA in CD138^+^ cells obtained from 4 HD, 11 MGUS, 133 MM patients, 9 PCL (GSE16122), and 23 HMCLs (GSE6205). (**B**) The expression of CX3CL1 protein was evaluated in eight purified BM CD138+ (**upper**) samples from four MM-ND and four MM-R patients and in seven HMCLs (**lower**) by western blot (positive control (Cnt+): Recombinant CX3CL1). (**C**) The western blot represents CX3CL1 protein expression on microenvironment cell lines. MM-ND—newly diagnosed MM; MM-R—relapsed MM; PCL—plasma cell leukemia; HMCLs—human myeloma cell lines; hTERT-MSC—human telomerase reverse transcriptase transduced mesenchymal stromal cell line; HBMESC—human BM-derived endothelial stem cells.

To unveil the source of the high BM CX3CL1 levels in MM patients, we analyzed *CX3CL1* mRNA expression by primary CD138^+^ cells and human myeloma cell lines (HMCLs) in public datasets (GSE16122) (GSE6205). Primary CD138^+^ cells and HMCLs expressed *CX3CL1* (Figure 3A) mRNA at low levels, with no statistical differences between HD, MGUS, MM, plasma cell leukemia (PCL), and HMCLs. Likewise, western blotting analysis revealed the absence of CX3CL1 protein in primary CD138^+^ cells from four newly diagnosed MM (MM-ND) and four relapsed MM (MM-R) patients (Figure 3B upper panel) and in seven HMCLs (JJN3, OPM2, U266, INA6, XG1, MM1S, and RPMI 8226) (Figure 3B lower panel).

Subsequently, we investigated the expression of CX3CL1 by osteoblast, osteocyte, mesenchymal, and EC lines and, as reported in Figure 3C, we showed a lack of CX3CL1 protein expression by western blot analysis. On the other hand, in our experiment setting and according to literature data [27,28], human umbilical vein endothelial cells (HUVEC) produced high levels of CX3CL1 protein when stimulated with the pro-inflammatory cytokines, tumor necrosis factor alpha (TNFα) (50 ng/ml) and interferon gamma (IFNγ) (10 ng/ml), for 24 h (Figure 3C).

**Figure 4:**
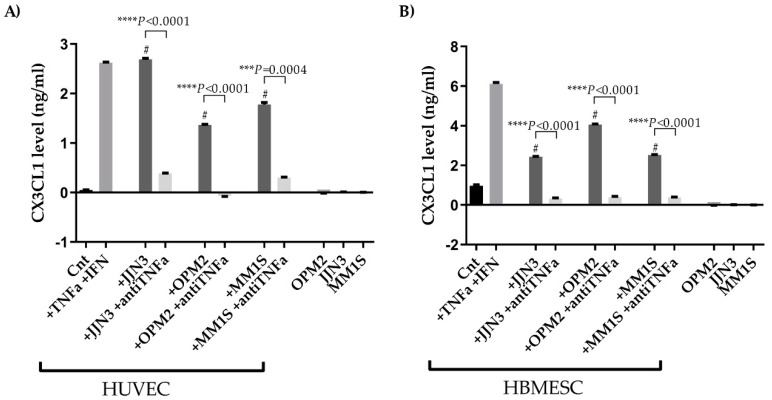
MM cells increase the angiogenic activity of ECs through CX3CL1 production. HUVEC (**A**) and HBMESC (**B**) were treated for 24 h with TNFα (50 ng/ml) and IFNγ (10 ng/ml), as positive control, and with CM of HMCLs (JJN3, OPM2, and MM1S) in presence or absence of anti-TNFα neutralizing antibody (2.7 µg/ml). At the end of the experiments, CX3CL1 levels were evaluated by enzyme-linked immunosorbent assay (ELISA) in the supernatants. The histograms represent the mean ± SD of CX3CL1 levels in two different experiments. (ECs + CM HMCLs vs. Cnt (HUVEC or HBMESC alone): ^#^ *P* < 0.05). ^#^ *P* values were calculated by two-tailed Student’s *t*-test. HUVEC—human umbilical vein endothelial cells; TNFα—with tumor necrosis factor alpha; IFNγ—interferon gamma.

It is well known that MM cells produced pro-inflammatory cytokines, such as TNFα [29]. For this reason, we explored the ability of MM cells to increase the production of CX3CL1 by treating ECs with the conditioned media (CM) of the HMCLs (OPM2, JJN3, and MM1S). We firstly showed that neither ECs nor HMCLs produce detectable levels of soluble CX3CL1 after 24 h (Figure 4A). Interestingly, the treatment of ECs with the CM of HMCLs significantly increased the production of soluble CX3CL1 compared to HUVECs and human BM-derived endothelial stem cells (HBMESCs) alone, as shown in Figure 3A (HUVEC + CM JJN3 vs. control (Cnt) *P* < 0.0001; HUVEC + CM OPM2 vs. Cnt *P* < 0.0001; HUVEC + CM MM1S vs. Cnt *P* = 0.0003, Student’s *t*-test) and Figure 4B (HBMESC + CM JJN3 vs. Cnt *P* = 0.0007; HBMESC + CM OPM2 vs. Cnt *P* = 0.0002; HBMESC + CM MM1S vs. Cnt *P* = 0.0006, Student’s *t*-test). In the same experimental setting, the treatment with anti-TNFα neutralizing antibody (2.7 µg/ml) for 24 h significantly inhibited the production of CX3CL1 by ECs induced by CM HMCLs. (HUVEC: CM JJN3 vs. CM JJN3 + anti-TNFα *P* < 0.0001; CM OPM2 vs. CM OPM2 + anti-TNFα *P* < 0.0001; CM MM1S vs. CM MM1S + anti-TNFα *P* = 0.0004, Student’s *t*-test) (HBMESC: CM JJN3 vs. CM JJN3 + anti-TNFα *P* < 0.0001; CM OPM2 vs. CM OPM2 + anti-TNFα *P* < 0.0001; CM MM1S vs. CM MM1S + anti-TNFα *P* < 0.0001, Student’s *t*-test) (Figure 4A,B).

### 2.4. CX3CR1 is Expressed by Endothelial Cells

**Figure 5:**
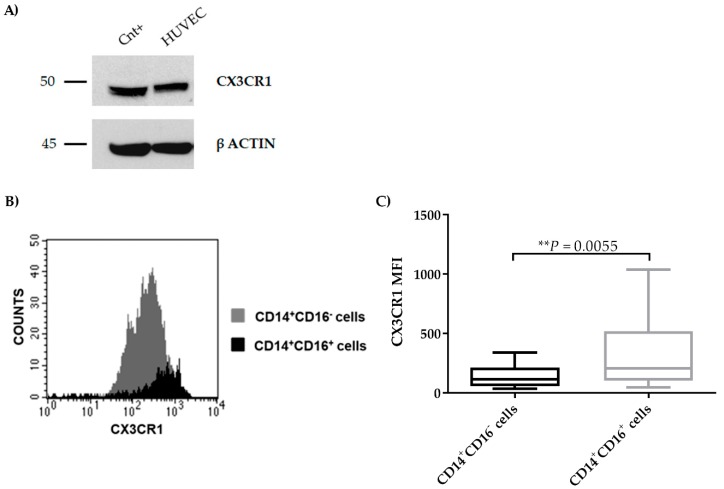
CX3CR1 expression by ECs. (**A**) Protein expression of CX3CR1 was evaluated by western blot on HUVEC cells (positive control: THP-1 cells). (**B**) The flow-cytometry histogram shows CX3CR1 expression and median fluorescence intensity (MFI) of CD14^+^CD16^+^ (black) and CD14^+^CD16^−^ (grey) monocyte subsets in one representative Multiple Myeloma patient (MM)-68. (**C**) Box plots represent the median level of CX3CR1 MFI in BM CD14^+^CD16^+^ and CD14^+^CD16^−^ monocytes from 2 MGUS, 4 SMM, and 17 MM patients (*P* value calculated by Mann–Whitney test).

Since the ECs produce CX3CL1 after pro-inflammatory *stimuli* from MM cells, we explored the CX3CR1 expression by MM cells and microenvironment cells. We found that HUVECs expressed CX3CR1 at protein levels (Figure 5A). In addition, BM monocytes from MM patients expressed CX3CR1, and, in particular, the subset CD14^+^CD16^+^ showed a higher expression of the receptor as compared to the CD14^+^CD16^−^ one (*P* = 0.0055, Mann–Whitney test) evaluated as median fluorescence intensity (MFI) by flow cytometry (Figure 5B,C).

### 2.5. CX3CL1/CX3CR1 Axis is Involved in Both In Vivo and In Vitro Multiple Myeloma-Induced Angiogenesis

**Figure 6:**
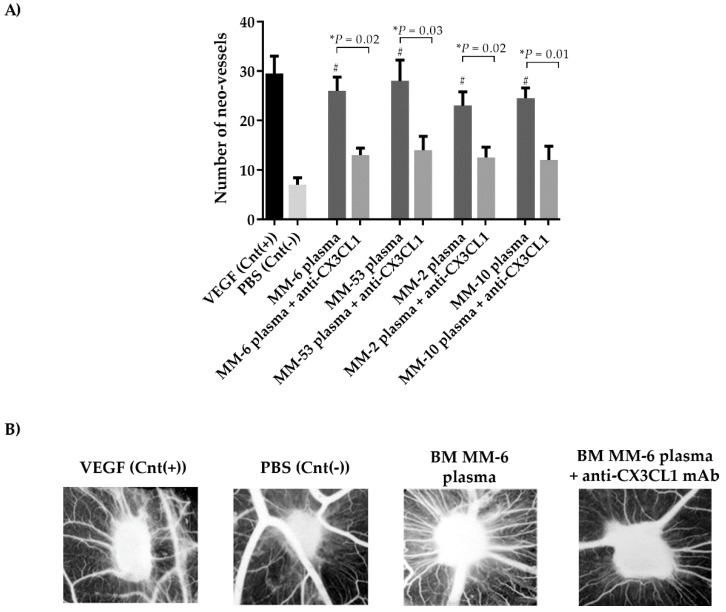
BM plasma from MM patients stimulates angiogenesis in vivo. The chorioallantoic membrane (CAM) assays were performed by treating the chicken chorioallantoic membrane for 12 days with BM plasma from four MM patients with or without anti-CX3CL1 (vascular endothelial growth factor (VEGF)) as positive control and phosphate-buffered saline (PBS) as negative control. (**A**) The histogram represents the mean ± SD of number of neo-vessels in CAM treated with BM plasma from four MM patients and with or without anti-CX3CL1 (0.3 μg/mL). (BM plasma vs. negative control: ^#^ *P* < 0.05). ^#^ *P* values were calculated by one-tailed Student’s *t*-test. (**B**) Representative macroscopic pictures of gelatin implanted on the top of chick embryo chorioallantoic membrane of one experiment using BM plasma from MM-11 patient with or without anti-CX3CL1. Original magnification 50×.

The potential involvement of CX3CL1 in MM-induced angiogenesis was investigated in vivo by chorioallantoic membrane (CAM) assay. As expected, BM plasma from four MM patients induced a significant increase of neo-vessel numbers compared to the negative control MM-11: BM plasma vs. negative control *P* = 0.0068; MM-22: BM plasma vs. negative control *P* = 0.011; MM-4: BM plasma vs. negative control *P* = 0.009; MM-18: BM plasma vs. negative control *P* = 0.005, Student’s *t*-test). The treatment with blocking anti-CX3CL1 monoclonal antibody (mAb) significantly reduced this pro-angiogenic activity (MM-6: BM plasma vs. anti-CX3CL1 *P* = 0.02; MM-53: BM plasma vs. anti-CX3CL1 *P* = 0.03; MM-2: BM plasma vs. anti-CX3CL1 *P* = 0.02; MM-10: BM plasma vs. anti-CX3CL1 *P* = 0.01, Student’s *t*-test) (Figure 6A). Figure 6B shows a representative picture of the CAM assay.

**Figure 7:**
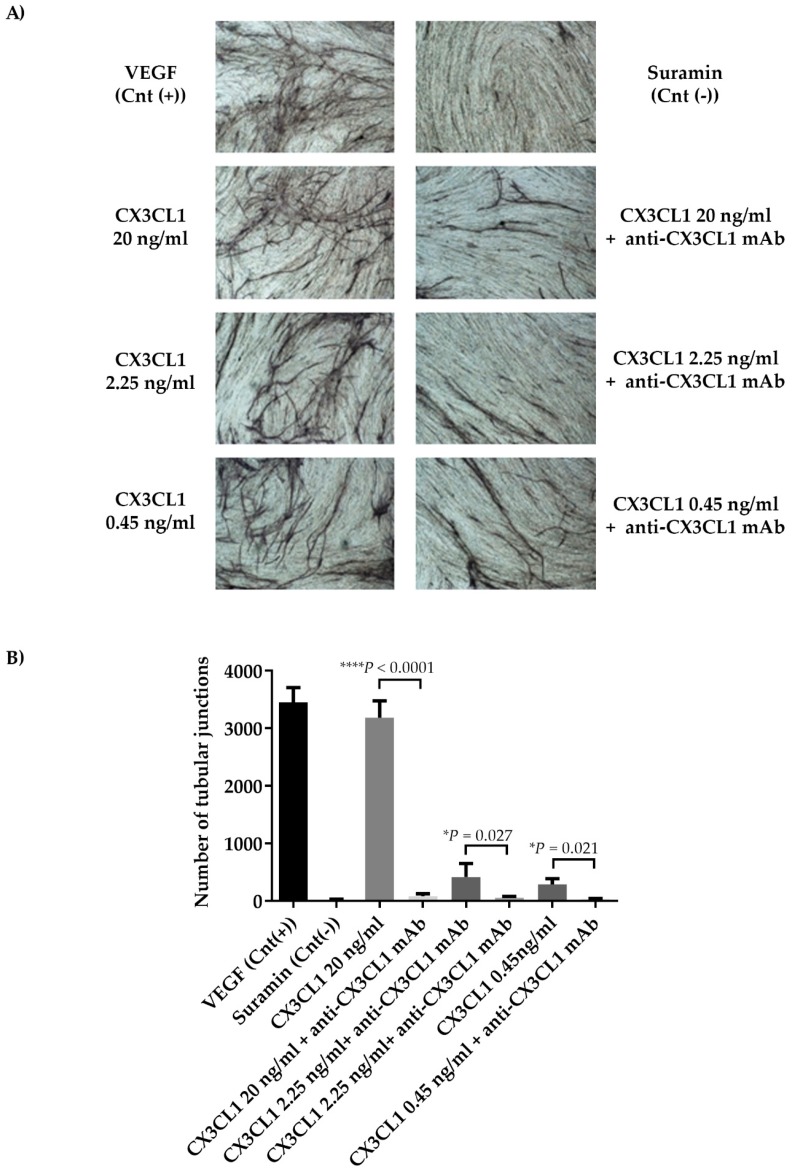
(**A**) In vitro angiogenic property of CX3CL1. The Angiokit co-culture of HUVEC and fibroblast cells was stimulated for 14 days with the recombinant human (rh) CX3CL1 at different concentrations (ranging 0.45–20 ng/ml) in the presence or absence of blocking anti-CX3CL1 monoclonal antibody (mAb) (0.6 µg/ml) (positive control: V2a Growth Medium added with VEGF; negative control: V2a Growth Medium added with suramin). (**B**) The graph represents the mean ± SD values of the number of tubular junctions of two replicates (*P* values were calculated by two-tailed Student’s *t*-test) quantified with Angioquant software.

On the basis of these results, we then performed an in vitro angiogenesis assay by testing different concentrations of recombinant human (rh) CX3CL1. For this aim, rhCX3CL1 was used at 20 ng/ml, which is known to be the maximum dose able to induce tube formation in HUVEC cells [30], and at concentrations comparable to the lowest (0.45 ng/ml) and the highest (2.25 ng/ml) found in our BM samples, in the presence or absence of blocking anti-CX3CL1 mAb (Figure 7A). For all concentrations, the presence of blocking anti-CX3CL1 mAb completely inhibited the ability of CX3CL1 to increase the number of tubular junctions (mean ± SD: rhCX3CL1 20 ng/ml 3186 ± 292.3 vs. anti-CX3CL1 mAb 80 ± 45.8, *P* < 0.0001; rhCX3CL1 2.25 ng/ml 418.25 ± 233.08 vs. anti-CX3CL1 mAb 55.5 ± 22.8, *P* = 0.027; rhCX3CL1 0.45 ng/ml 288.75 ± 99.72 vs. anti-CX3CL1 mAb 33 ± 9.8*P* = 0.021, Student’s *t*-test) (Figure 7B). All these data confirm the direct involvement of CX3CL1 in MM-induced angiogenesis.

## 3. Discussion

The expression of the CX3CL1/CX3CR1 axis in MM cells and their microenvironment was defined in this study. We showed that higher levels of soluble CX3CL1 were present in the BM plasma of MM patients as compared with indolent monoclonal gammopathies. Moreover, we found that the CX3CL1/CX3CR1 axis was mainly involved in the increased BM vascularization and, consequently, in MM-induced angiogenesis.

CX3CL1, also known as fractalkine, is a chemokine found either as a membrane-bound protein or in a soluble form after cleavage by the metalloproteases ADAM10 [12] and ADAM17 [13]. All the biological effects of CX3CL1 are the results of the binding to its receptor CX3CR1 [14]. CX3CL1 is a chemokine that combines chemoattractant and pro-adhesion properties by recruiting cells that express CX3CR1; CX3CL1 exhibits pro-inflammatory properties by its chemotactic activity for NK cells, DCs, monocytes, and mature osteoclasts [31,32]. In addition, soluble CX3CL1 is involved in angiogenesis and endothelial cell chemotaxis [33,34]. CX3CL1 displays pro-angiogenic and pro-inflammatory effects in a number of pathological conditions, including rheumatoid arthritis, diabetes, and cancers [35,36]. Indeed, the CX3CL1/CX3CR1 axis is involved in the interaction between tumor cells and the microenvironment by the regulation of tumor cell invasion, migration, and adhesion and promotes bone metastasis [19,20,21,22].

Multiple Myeloma is typically characterized by a pro-inflammatory and pro-angiogenic microenvironment that supports MM cell growth and the progression of the disease [37]. However, the role of the CX3CL1/CX3CR1 axis in MM has not yet been explored. Our data indicated the CX3CL1 progressively increased across the monoclonal gammopathies and correlated with the BM MVD and PC infiltration but not with the presence of osteolytic bone disease. Wada et al. described that few HMCLs expressed CX3CR1 and that CX3CL1-treated HMCLs increased osteoclast differentiation [23] accordingly with the role of CX3CL1 in bone resorption [31]. Clearly, the lack of relationship between CX3CL1 BM plasma levels and the presence of bone disease in MM patients excludes the possible role of this chemokine system in MM-induced osteoclastogenesis as reported for other pro-osteoclastogenic cytokines [38]. However, our data clearly indicated that both HMCLs and primary CD138^+^ PCs did not express *CX3CR1* mRNA. Consistently, gene and protein expression analysis revealed the absence of CX3CL1 expression by PCs. Our data also indicated that ECs may contribute to the higher BM CX3CL1 levels in MM patients. The levels of BM CX3CL1 positively correlated with both the number of CD34^+^ vessels and with the percentage of BM PCs in bone biopsies. In agreement with what was reported by others [39,40], ECs stimulated with pro-inflammatory *stimuli* produced high levels of soluble CX3CL1. In the same way, ECs showed high CX3CL1 production after treatment with CM of several HMCLs supporting the notion that the cross talk between MM and the microenvironment cells stimulated the production of inflammatory cytokines. Indeed, it is well reported that MM cells secreted TNFα [29]. Our in vitro data showed that the treatment with anti-TNFα neutralizing antibody inhibited the MM-mediated production of CX3CL1 indicating the involvement of this well-known pro-inflammatory cytokine. The role of TNFα in the pathophysiology of MM is well established as being a pro-survival factor of MM cells involved in osteoclast activation and angiogenesis [29]. Our study suggests that the pro-angiogenic effect of the MM-derived TNFα could be also mediated by the up-regulation by ECs of CX3CL1 that, in turn, stimulated new vessel formation.

Among the different microenvironment cell types, we then confirmed that ECs expressed CX3CR1. Previous data demonstrated that the angiogenic property of CX3CL1 was mediated by the phosphorylation of extracellular signal-regulated kinases (ERK), Protein kinase B (Akt), and endothelial nitric oxide (NO) synthase (eNOS), as well as an increase in NO production [30]. CX3CL1 also stimulated EC proliferation, migration, and tube formation in vitro and in vivo [41].

Our data in MM patients indicated that, other than the relationship between BM plasma CX3CL1 levels and BM MVD, a statistical correlation between CX3CL1 and the number of BM CD14^+^CD16^+^ also exists. In a multivariate analysis, we demonstrated that BM CX3CL1 plasma levels correlated with the number of BM CD14^+^CD16^+^. We and others previously reported higher levels of this subpopulation of monocytes in MM patients as compared to MGUS patients that are able to generate osteoclasts in vitro [24,42,43]. There are many data reporting the pro-angiogenic role of CD14^+^CD16^+^ non-classical monocytes either in physiological conditions or in the tumor microenvironment [44]. Moreover, it has been reported that CX3CL1 may induce the pro-angiogenic profile of CD14^+^CD16^+^ monocytes [21,45] supporting the role of CX3CL1 in the angiogenic process in MM patients. The link between inflammation and angiogenesis is well established and the effect of CX3CL1 treatment on CX3CR1-positive monocytes to stimulate their angiogenic differentiation has been reported [18,21].

Thus, based on the relationship between CX3CL1 soluble levels with the angiogenic properties of MM patients, we next explored the contribution of the CX3CL1/CX3CR1 axis in MM-induced angiogenesis in pre-clinical models. The pro-angiogenic role of CX3CL1 has been investigated in two different models both in vivo and in vitro. In CAM assay, as expected, BM plasma from MM patients increased the neo-angiogenesis effect that was inhibited by the treatment with blocking anti-CX3CL1 mAb. Similarly, rhCX3CL1 stimulated tube formation at concentrations comparable to those found in MM plasma whereas the addition of blocking anti-CX3CL1 mAb inhibited this effect. Overall, these data demonstrated the role of this system in the pro-angiogenic properties of MM cells through the interaction with ECs and the activation of the CX3CL1/CX3CR1 axis.

Our data support the possible role of this system as a potential therapeutic target in MM. Currently, there are no data reporting the use of modulators of the CX3CL1/CX3CR1 axis as anti-angiogenic drugs. However, the anti-inflammatory effect of blocking CX3CL1/CX3CR1 antagonists has been previously reported [46]. A humanized anti-CX3CL1 mAb E6011 has been tested in a phase 1/2 clinical trial in patients with rheumatoid arthritis showing good tolerability and safety [47]. Specifically, it targets the trafficking of immune cells, which produce pro-inflammatory cytokines in the local inflamed sites [47]. Others studies suggested that anti-CX3CL1 mAbs inhibited the migration of CX3CR1+ macrophages and cytotoxic effector T cells responsible for the production of pro-inflammatory cytokines suggesting their potential role in blocking the inflammation cascade in the local inflamed region [46]. Our data on the role of CX3CL1 in MM-induced angiogenesis support the possible use of the E6011 in MM patients to block the pro-angiogenic and pro-inflammatory activity of CXC3CL1. As known, drugs with anti-angiogenic and anti-inflammatory activity such as thalidomide and proteasome inhibitors are widely used with success in the treatment of MM patients. Interestingly, it was reported that Bortezomib inhibits CX3CL1 production in an inflammatory rat model [48].

In addition, the high-affinity small-molecule inhibitor of CX3CR1 (AZD8797) has been investigated in a rat model of multiple sclerosis where it blocked the infiltration of CX3CR1-expressing cells into the central nervous system [49]. More recently a novel small-molecule CX3CR1 antagonist was developed and tested in a preclinical breast cancer model showing an anti-bone metastatic effect [50]. Our evidence showing the involvement of CX3CL1 in MM-induced angiogenesis, together with the pro-inflammatory bone microenvironment that characterized MM patients [51], gives the rationale to test this possible therapeutic approach by blocking the CX3CL1/CX3R1 axis in MM also.

## 4. Materials and Methods

### 4.1. Patient Samples

A total cohort of 111 patients (56 males and 55 female) with PC disorders were included in the study until September 2018: 16 patients with MGUS (median age: 66 years; range: 42–78), 25 with SMM (median age: 69 years; range: 38–93), and 70 with active MM (median age: 73 years; range: 52–89) including 49 MM-ND and 21 MM-R.

Ten HD were used as controls. All patients were diagnosed according to the International Myeloma Working Group (IMWG) revised criteria [52]. Patients were considered to have bone involvement based on the presence of one or more osteolytic lesions and/or osteoporosis in the skeletal survey, according to the hyperCalcemia, Renal failure, Anemia, Bone lesions (CRAB) criteria. The presence of three or more osteolytic lesions or fractures defined HBD. Patients with a negative skeletal survey or a positive skeletal survey with fewer than three osteolytic lesions were considered to have LBD [38]. The main clinical characteristics of all the patients enrolled in the study are summarized in Supplemental Table S1.

Bone Marrow aspirates (5 + 5 ml, treated with EDTA to prevent clotting) and bone biopsies were obtained from the iliac crest of each patient. BM plasma was collected after centrifugation, and stored at −20 °C until the analysis. Patient samples were obtained after informed consent, according to the Declaration of Helsinki. The study was included in a project approved by the Institutional Ethical Review Board of Parma Hospital (0006639-6.2.2 of 22 February 2017).

Bone marrow mononuclear cells (MNCs) were obtained after gradient centrifugation with Ficoll solution (Lympholyte^®^ Cell Separation Media, CEDARLANE, ON, Canada). CD138^+^ PCs were isolated from BM MNCs by an immunomagnetic method with anti-CD138 antibody conjugated with microbeads (Miltenyi Biotec, Bergisch Gladbach, Germany) from MM patients, as previously described [53].

### 4.2. Immunohistochemistry

Bone biopsy sections were incubated with mouse anti-human CD138 clone B-A38 (ready to use, Ventana Medical Systems, Tucson, AZ, USA) and the reaction was revealed with polymeric ultraView Universal DAB detection (Ventana Medical Systems) or with rabbit anti-human CD34 (1:100, Santa Cruz Biotechnology, Santa Cruz, CA, USA). After washing, sections were incubated with a secondary antibody (rat anti-Immunoglobulin G horseradish peroxidase (HRP); Millipore, Burlington, MA, USA; 1:250) and the reaction was revealed with a solution of 3,3′-diaminobenzidine tetrahydrochloride (Liquid DAB Substrate Chromogen System, DAKO, Glostrup, Denmark). MVD was evaluated as the number of CD34^+^ vessels/mm^2^ in MM patients’ bone biopsies. Images of IHC analyses were captured by a DP22 digital camera (Olympus, Hamburg, Germany) and analyzed with the OLYMPUS Stream software, adjusting tone and contrast to ensure the best image quality.

### 4.3. Flow-Cytometry Assay

After MNC isolation, cells were collected and analyzed by flow cytometry using the following mAbs (BD Biosciences, Milan, Italy): Anti-CD138 R-phycoerythrin (PE) (552026), anti-CD14 Allophycocyanin (APC) (cod. 555399), anti-CD16 fluorescein isothiocyanate (FITC) (cod. 555406), and anti-CX3CR1 PE (cod. 565796). The analyses were performed on a two-laser FACSCalibur instrument (BD Biosciences) using CellQuest software v.4 (BD Biosciences).

### 4.4. Analysis of Gene Expression Profiles

The gene expression profiles of CX3CL1 were evaluated in PCs of 4 HD, 11 MGUS, 133 MM, 9 PCL patients (GSE16122), and 23 HMCLs (GSE6205). The data were extracted from CEL files using a robust multi-array average (RMA) normalization procedure and custom chip definition file (CDF) annotation package (GeneAnnot v2.2.1, Rehovot, Israel), as previously described [54].

### 4.5. Cells and Cell Culture Conditions

#### 4.5.1. Cell Lines

The HMCLs JJN3, OPM2, U266, MM1S, NCI-H929, RPMI-8226, and the acute monocytic leukemia (THP-1) cell lines were purchased from Leibniz Institute Deutsche Sammlung von Mikroorganismen und Zellkulturen GmbH (Braunschweig, Germany). The HMCLs XG1 and INA6 were obtained by the Laboratory of INSERM from Dr. Martine Amiot (Nantes, France).

The human bone marrow stromal cell line, HS-5, was purchased from ATCC (Manassas, VA, USA). The human telomerase reverse transcriptase transduced mesenchymal stromal cell line (hTERT-MSCs) was kindly gifted from Dr. Giuseppe Gaipa (Monza, Italy). Immortalized human osteoblast-like (HOBIT) cells were kindly provided from Dr. B. L. Riggs (Rochester, MN, USA). The human pre-osteocytic cells (HOB-01) were established from human bone and kindly provided by Julia Billars (Collegeville, PA, USA). HUVEC were obtained by ATCC. The human BM derived endothelial stem cells (HBMESC) were purchased from Celprogen (Torrance, CA, USA) and both were maintained in culture with HBMESC complete growth media with serum (Celprogen) on a flask pre-coated with collagen type 1 (Roche, Basel, Switzerland). All cell lines were authenticated and tested for mycoplasma contamination.

#### 4.5.2. Cell Conditions and Experimental Procedures

CM from HMCLs were collected after 48 h of culture and stored at −20 °C until use. In collagen-coated six-well plates, HUVEC and HBMESC were incubated in the presence or absence of diluted CM (1:3) of OPM2, JJN3, and MM1S for 24 h and with or without recombinant TNFα (50 ng/ml) (OriGene; Rockville, MD, USA) and/or neutralizing anti-TNFα mAb (2.7 µg/ml able to neutralize 50 ng/ml) (R&D systems Minneapolis, MN, USA) for 24 h. HUVEC and HBMESC treated with TNFα (50 ng/ml) and IFNγ (10 ng/ml) (Sigma–Aldrich, Italia, Milan, Italy) for 24 h were used as a positive control. At the end of the experiment, the culture media were collected and analyzed by enzyme-linked immunosorbent assay (ELISA).

### 4.6. ELISA

Soluble CX3CL1 was measured by ELISA (R&D System, Minneapolis, MN, USA) in BM plasma samples and in HMCLs, HUVEC, HBMESC, and co-cultured CM, according to the instructions of the manufacturer.

### 4.7. Western Blotting Analysis

Western blotting was performed on the HMCLs (JJN3, OPM2, U266, INA6, XG1, MM1S, and RPMI-8226) and on primary CD138^+^ cells purified from four MM-ND and four MM-R patients, as previously described [55]. Blots were incubated at 4 °C overnight with the following antibodies: Anti-CX3CL1 (rabbit, polyclonal, 1:1000) and anti-β-actin (mouse, monoclonal 1:5000) (Sigma–Aldrich) as internal control.

### 4.8. Chick Embryo Chorioallantoic Membrane Assay

Fertilized White Leghorn chicken eggs were incubated at 37 °C in constant humidity. On day 3 of incubation, a square window was cut in the shell of each egg, and 2–3 ml of albumen was removed to allow detachment of the developing CAM. The window was sealed with a glass coverslip, and the eggs were returned to the incubator. On day 8 of incubation, the coverslips were removed and the growing CAMs (10 eggs per group) were treated with 1 lL PBS (negative control); 1 µl PBS with 250 ng vascular endothelial growth factor (VEGF) (R&D Systems, positive control); 1 µl MM BM plasma treated or not with a neutralizing antibody to CX3CL1 0.3 µg/ml (R&D Systems). The coverslips were replaced after these treatments, and the CAMs were examined daily until day 12 of incubation, when they were photographed in *ovo* using a stereomicroscope equipped with a camera and image analysis system (Olympus Italia, Italy). Blood vessels entering the sponges within the focal plane of the CAM were counted by two observers in a double-blind fashion at a magnification of 50×.

### 4.9. Angiogenesis In Vitro Assay

In vitro angiogenesis was assessed by a V2a angiogenesis assay kit obtained from Cellworks (Buckingham, UK). Endothelial cells were stimulated with VEGF (2 ng/ml, positive control) or suramin (20 μM, negative control) or with recombinant CX3CL1 (20 ng/ml, 2.09 ng/ml, or 0.45 ng/ml) or with a neutralizing antibody to CX3CL1 (0.6 µg/ml) (R&D Systems). At day 14, cells were fixed and stained using an anti-CD31 Ab provided with the V2a angiogenesis assay kit following the manual instructions. Angioquant software was used to quantify the number and length of the formed tubules and the number of junctions.

### 4.10. Statistical Analysis

Comparisons among the three groups were made with the Kruskal–Wallis test and pairwise comparisons with the Mann–Whitney test or with parametric unpaired t-test. The Spearman test was used for correlations. Relationships adjusted for potential covariates were examined by multiple linear regression. A *P* value <0.05 was considered significant. GraphPad Prism 6 software was used for all the statistical analyses.

## 5. Conclusions

In conclusion, our data indicate that BM CX3CL1 plasma levels are increased across the progression of monoclonal gammopathies from MGUS to MM and that the high CX3CL1 BM levels in MM correlated with the presence of a high grade of vascularization. The major source of CX3CL1 seems to be ECs that release CX3CL1 in the presence of MM cells with the involvement of the pro-inflammatory cytokine TNFα. By both in vitro and ex vivo angiogenic models we finally demonstrated the role of the CX3CL1/CX3R1 axis in the pro-angiogenic switch induced by MM cells. Our results underline the importance of the BM CX3CL1 levels as a possible new biomarker for the progression of MM related to the increase of the BM vascularization. The role of the CX3CL1/CX3CR1 axis as a new possible anti-angiogenic therapeutic target in MM is also suggested by our results.

## Supplementary Materials

The following are available online at https://www.mdpi.com/2072-6694/11/3/321/s1, Figure S1: Evaluation of CX3CL1 bone marrow levels in myeloma patients with or without bone disease. Table S1: Clinical characteristics of patients.
