# Supplementary material for: Bone Marrow CX3CL1/Fractalkine is a New Player of the Pro-Angiogenic Microenvironment in Multiple Myeloma Patients
Source: Cancers (Basel). 2019 Mar 6;11(3):321. doi: 10.3390/cancers11030321 (PMC6469019; doi:10.3390/cancers11030321)

SUPPLEMENTAL FIGURE S1: Evaluation of CX3CL1 bone marrow levels in myeloma patients with or without bone disease. BM CX3CL1 plasma levels in multiple myeloma patients (A) with (W) or without (W/O) osteolytic lesions (*P* = 0.34, not significant (NS)) or (B) the presence of high bone disease (HBD) compared to low bone disease (LBD) (*P* = 0.78, NS).

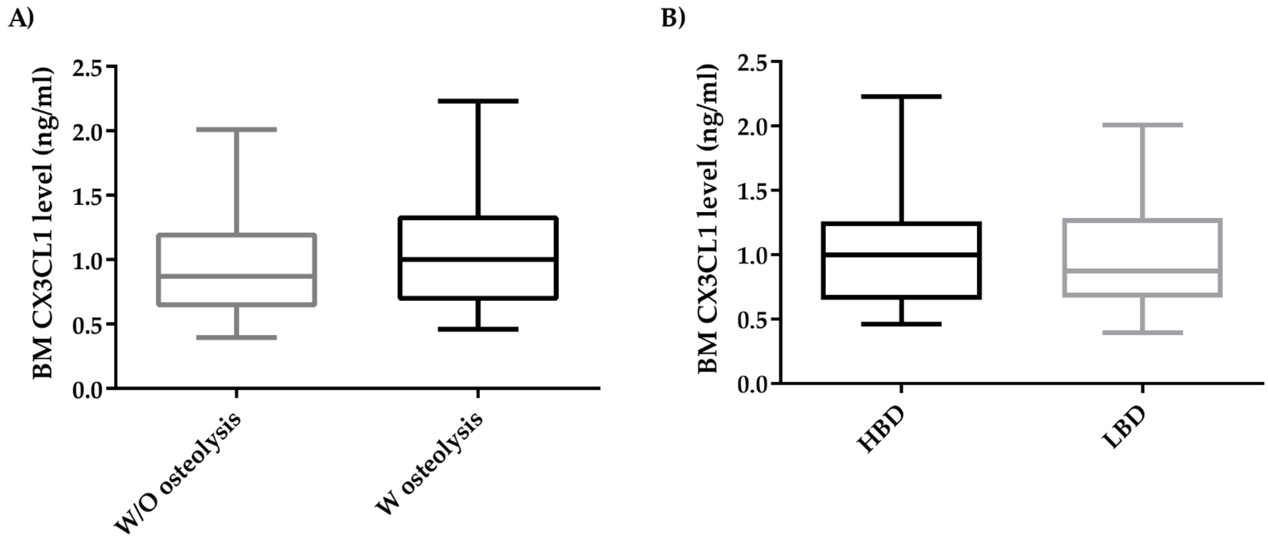

Supplement: Supplementary file 1 [file cancers-11-00321-s001.zip › SUPPLEMENTAL FIGURE S1.pdf]
