# Supplementary material for: Bone Marrow CX3CL1/Fractalkine is a New Player of the Pro-Angiogenic Microenvironment in Multiple Myeloma Patients
Source: Cancers (Basel). 2019 Mar 6;11(3):321. doi: 10.3390/cancers11030321 (PMC6469019; doi:10.3390/cancers11030321)
Supplement: Supplementary file 1 [file cancers-11-00321-s001.zip › SUPPLEMENTAl DATA.pdf]

Supplemental Table S1: Clinical Characteristc of patients

DIAGNOSIS	STAGE	ISS	GENDER	AGE	LIGHT CHAINS	OSTEOLYSIS	HBD/LBD
HD-1			F	34	-	-	-
HD-2			M	68	-	-	-
HD-3			M	66	-	-	-
HD-4			F	66	-	-	-
HD-5			M	64	-	-	-
HD-6			M	64	-	-	-
HD-7			M	74	-	-	-
HD-8			M	74	-	-	-
HD-9			M	69	-	-	-
HD-10			M	54	-	-	-
MGUS-1			M	72	k	-	-
MGUS-2			F	43	l	-	-
MGUS-3			M	42	k	-	-
MGUS-4			M	66	l	-	-
MGUS-5			M	78	k	-	-
MGUS-6			F	54	l	-	-
MGUS-7			M	77	k	-	-
MGUS-8			M	43	k	-	-
MGUS-9			F	72	k	-	-
MGUS-10			M	46	l	-	-
MGUS-11			M	57	k	-	-
MGUS-12			M	67	k	-	-
MGUS-13			M	61	-	-	-
MGUS-14			F	55	k	-	-
MGUS-15			F	77	k	-	-
MGUS-16			M	73	k	-	-
SMM-1			F	38	k	-	-
SMM-2			F	68	k	-	-
SMM-3			F	67	l	-	-
SMM-4			F	69	l	-	-
SMM-5			M	76	k	-	-
SMM-6			M	58	k	-	-
SMM-7			F	64	k	-	-
SMM-8			F	41	k	-	-
SMM-9			F	51	l	-	-
SMM-10			F	70	k	-	-
SMM-11			M	83	k	-	-
SMM-12			M	-	-	-	-

SMM-13			M	70	k	-	-
SMM-14			F	65	k	-	-
SMM-15			F	75	l	-	-
SMM-16			M	58	k	-	-
SMM-17			M	57	k	-	-
SMM-18			M	80	k	-	-
SMM-19			M	71	k	-	-
SMM-20			M	81	k	-	-
SMM-21			M	80	k	-	-
SMM-22			M	93	k	-	-
SMM-23			M	63	k	-	-
SMM-24			F	57	k	-	-
SMM-25			F	81	k	-	-
MM-1	ND	III	F	67	l	YES	LBD
MM-2	ND	II	M	65	l	NO	
MM-3	ND	II	M	55	k	YES	HBD
MM-4	ND	II	M	68	k	NO	
MM-5	ND	II	F	79	k	NO	
MM-6	ND	III	F	60	l	YES	HBD
MM-7	ND	I	M	62	k	NO	
MM-8	ND	III	F	75	l	YES	NA
MM-9	ND	III	F	72	k	YES	HBD
MM-10	ND	I	F	61	k	NO	
MM-11	ND	III	M	73	k	NO	
MM-12	ND	III	M	84	l	YES	LBD
MM-13	ND	III	F	73	l	YES	HBD
MM-14	ND	I	M	53	k	YES	HBD
MM-15	ND	II	F	70	k	YES	HBD
MM-16	ND	I	F	80	k	YES	HBD
MM-17	ND	II	F	88	l	NO	
MM-18	ND	III	M	58	k	YES	HBD
MM-19	ND	II	F	83	k	NO	
MM-20	ND	II	F	71	k	YES	HBD
MM-21	ND	III	M	69	l	YES	HBD
MM-22	ND	II	F	78	l	YES	NA
MM-23	ND	II	M	78	l	YES	HBD
MM-24	ND	I	F	62	k	YES	NA
MM-25	ND	I	M	76	k	YES	LBD
MM-26	ND	III	F	86	l	NO	
MM-27	ND	III	F	89	k	NO	
MM-28	ND	I	M	70	k	YES	HBD
MM-29	ND	III	F	73		NO	

MM-30	ND	II	M	67	k	NO	
MM-31	ND	III	M	88	k	YES	HBD
MM-32	ND	III	F	88	k	NO	
MM-33	ND	III	M	72	k	YES	HBD
MM-34	ND	III	M	76	l	YES	HBD
MM-35	ND	II	F	73	l	YES	HBD
MM-36	ND	II	F	71	k	NO	
MM-37	ND	II	F	52	k	NO	
MM-38	ND	III	M	85	k	YES	HBD
MM-39	ND	III	F	74	k	YES	NA
MM-40	ND	II	M	77	l	YES	HBD
MM-41	ND	III	M	79	l	NO	
MM-42	ND	III	F	86	l	YES	HBD
MM-43	ND	III	M	68	k	NA	
MM-44	ND	III	F	74	k	YES	NA
MM-45	ND	III	M	88	l	NA	
MM-46	ND	I	F	53	k	YES	HBD
MM-47	ND	II	F	79	l	YES	LBD
MM-48	ND	III	F	84	k	NA	
MM-49	ND	III	M	77	l	NA	
MM-50	R	I	F	77	l	NO	
MM-51	R	II	F	59	k	YES	HBD
MM-52	R	II	M	82	k	NA	
MM-53	R	III	M	73	l	YES	LBD
MM-54	R	III	M	80	k	NO	
MM-55	R	III	F	82	k	YES	HBD
MM-56	R	I	M	67	l	NO	
MM-57	R	I	F	70	k	YES	HBD
MM-58	R	I	F	84	k	NA	
MM-59	R	II	M	74	l	YES	HBD
MM-60	R	III	F	72	k	YES	NA
MM-61	R	II	F	71	k	YES	HBD
MM-62	R	I	F	67		NO	
MM-63	R	I	M	70	k	YES	HBD
MM-64	R	III	F	69	k	NO	
MM-65	R	III	F	84	k	NO	
MM-66	R	I	M	61	k	NO	
MM-67	R	I	F	65	l	NO	
MM-68	R	I	M	70	k	NO	
MM-69	R	III	M	63	l	NA	
MM-70	R	III	M	74	k	YES	HBD

Abbreviations: MGUS: Monoclonal Gammopathy of Undetermined Significance; SMM: Smoldering Multiple Myeloma; MM: Multiple Myeloma; ND: Newly Diagnosed; R: Relapsed; F: female; M: male; ISS: International Staging System; HBD: High Bone Disease; LBD: Low Bone Disease.
